# From blood to brain: blood cell-based biomimetic drug delivery systems

**DOI:** 10.1080/10717544.2021.1937384

**Published:** 2021-06-18

**Authors:** Yong-Jiang Li, Jun-Yong Wu, Jihua Liu, Xiaohan Qiu, Wenjie Xu, Tiantian Tang, Da-Xiong Xiang

**Affiliations:** a Department of Pharmacy, The Second Xiangya Hospital, Central South University, Changsha, China; b Hunan Provincial Engineering Research Centre of Translational Medicine and Innovative Drug, Changsha, China; c Institute of Clinical Pharmacy, Central South University, Changsha, China

**Keywords:** Blood brain barrier, blood cells, biomimetic, brain drug delivery

## Abstract

Brain drug delivery remains a major difficulty for several challenges including the blood–brain barrier, lesion spot targeting, and stability during circulation. Blood cells including erythrocytes, platelets, and various subpopulations of leukocytes have distinct features such as long-circulation, natural targeting, and chemotaxis. The development of biomimetic drug delivery systems based on blood cells for brain drug delivery is growing fast by using living cells, membrane coating nanotechnology, or cell membrane-derived nanovesicles. Blood cell-based vehicles are superior delivery systems for their engineering feasibility and versatile delivery ability of chemicals, proteins, and all kinds of nanoparticles. Here, we focus on advances of blood cell-based biomimetic carriers for from blood to brain drug delivery and discuss their translational challenges in the future.

## 1. Introduction

The treatment of brain diseases, including brain tumors (van Tellingen et al., 2015), stroke (Catanese et al., 2017), neurodegenerative disorders (Vissers et al., 2019), etc. has been challenging. The clinical approval success rate of drugs for brain diseases is one of the lowest compared to other fields (Miller, 2010; Gribkoff & Kaczmarek, 2017). The major difficulties restricting the clinical translation of drugs for brain diseases include the blood–brain barrier (BBB) (Stanimirovic et al., 2015; Xie et al., 2019); pharmacokinetic profile alteration (de Lange et al., 2017; Yamamoto et al., 2018); toxicity of drugs (Vuillemenot et al., 2016). The development of drug delivery systems holds great potential to endow BBB penetration ability, change the biodistribution and pharmacokinetics of drugs to improve the efficacy while reducing side effects (Reddy et al., 2021).

In recent years, emerging efforts have been dedicated to developing biomimetic drug − delivery systems by using complex natural biological components or mimicking the structure (Parodi et al., 2013; Hu et al., 2015; Fang et al., 2018). For delivery of drugs for brain diseases therapy, biomimetic drug delivery systems may help increase biocompatibility, long − term circulation and more importantly, penetrate the BBB to increase drug concentration at the target site (Chen et al., 2020). The most commonly developed cell-based vehicles for biomimetic drug delivery include living cells (Wang et al., 2015), cell membranes (Luk & Zhang, 2015), and nanovesicles (Usman et al., 2018), depending on the target of disease and cargo for delivery. Cell and cell membrane are similar vehicles for drug delivery by direct loading of drugs or coating drug nanoparticles. Cell and cell membrane coating are both effective for biointerfacing (Kroll et al., 2017). Properties cell membranes inherent from the source cells, bestowing a wide range of advantages for circulation and targeting. Cell-derived nanovesicles, such as exosomes, are natural carriers with intrinsic features including crossing various biological barriers, stability during circulation and potential targeting ability (Pegtel & Gould, 2019; Kalluri & LeBleu, 2020).

**Figure 1.**
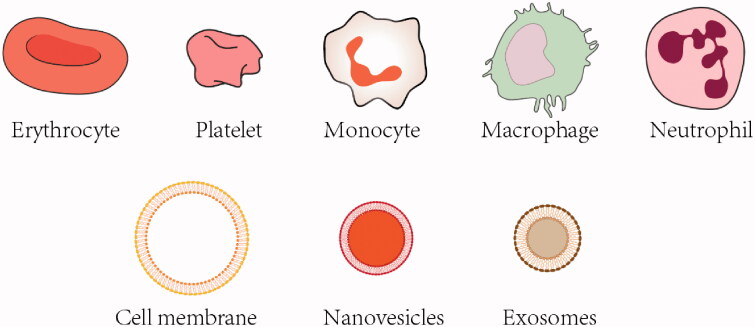
Different types of blood-cell based vehicles for brain drug delivery.

Despite a large number of cell-based carriers has been explored for drug delivery, blood cell-based vehicles have shown a variety of functions suitable for brain drug delivery (Sun et al., 2017). Specific properties of blood cell-based delivery systems include high biocompatibility, long circulation, targeted drug delivery, and suitability for a wide range of cargoes (Sun et al., 2017). Since blood cell-based delivery systems hold great promises for facilitating the development of therapeutics for brain diseases therapy from blood-to-brain, we introduce challenges for brain drug delivery and summarize the recent progress on the study of blood cell-based delivery systems for brain drug delivery (Table 1). Cell types in the blood include erythrocytes, platelets, and leukocytes. Here, we focus on these blood cells and their derived membranes or vesicles as delivery vehicles (Figure 1). A specific emphasis is placed on the different features of blood cells. The advantages of specific cells for different brain disease applications are covered in depth. Finally, future directions and challenges of the utility of blood cell-based delivery systems for brain drug delivery are discussed.

**Table 1.** Summary of blood cell-based vehicles for brain drug delivery.

Source of blood cells	Vehicles	Cargo	Strategies	Brain disease and model	Key advances	Ref.
Erythrocyte	Erythrocyte membrane	Dox-loaded PLGA nanoparticles	Neurotoxin-derived peptide for brain targeting Erythrocyte membrane for long circulation DOX-PLGA for drug release	Glioma	Brain targeting effect, improved survival of glioma-bearing mice and reduced toxicity	Chai et al. (2017)
	Erythrocyte membrane	Docetaxel nanocrystals	RGD peptide for tumor targeting Cell membrane for long circulation DOX-PLGA for tumor killing	Glioma	Superior tumor accumulation and enhanced therapeutic efficacy	Chai et al. (2019)
	Erythrocyte membrane	miR155-bearing nanogel	Cell membrane prolongs the circulation lifetime M2pep peptide for active targeting miR155 nuclei acid for promotion of cytokine production and shift macrophage and microglia polarization	Glioblastoma	Active tumor-targeting capability, reprograming of microglia and macrophages from M2 to M1 phenotype, excellent tumor inhibition efficacy and prolonged survival	Gao et al. (2021)
	Erythrocyte membrane	Manganous tetroxide nanoparticles	Erythrocyte membrane for high stability, long circulation, and BBB penetration Mn_3_O_4_ nanoparticles as antioxidant mimic nanoenzyme T7 peptides for enhancing BBB penetration	Ischemic stroke/MCAO rats	Distinct therapeutic effect for rescuing neurocytes before and after thrombolysis by oxygen sponge effect and scavenging free radicals	Shi et al. (2020)
Platelet	Platelet membrane	ZL006e-loaded dextran derivative polymeric nanoparticle	Recombinant tissue plasminogen activator for thrombin-triggering Tat peptide for BBB penetration Platelet membrane for long circulating and thrombus targeting ZL006e for neuroprotection	Ischemic stroke/MCAO rats	Enhanced anti-ischemic stroke efficacy by recovering the cerebral blood supply and protecting neurons of ischemic penumbra	Xu et al. (2019)
	Platelet nanobubbles	None	Platelet nanobubbles for accurate lesion-targeting and microvascular bio-remodeling of the stroke lesion	Photothrombotic stroke mice	Stroke lesion microvasculature theranostics and reflecting the dynamic structural nature of the recanalization process	Li et al. (2018)
	Platelet membrane	l-Arginine and γ-Fe_2_O_3_ magnetic nanoparticles	Platelet membranes for long-term stability and targeting to the damaged blood vessel γ-Fe_2_O_3_ nanoparticles for magnetic targeting l-arginine for producing NO	Focal cerebral ischemia mice model	Rapid targeting to ischemic stroke lesions under the guidance of an external magnetic field *In situ* generation of NO for enhanced vasodilation, recovery of blood flow, and reperfusion of the stroke microvascular	Li et al. (2020b)
Leukocytes	Monocyte	DOX-loaded nanodiamonds	Monocytes for BBB penetration and GBM infiltration	Glioblastoma	Improved tumor drug delivery efficacy and damage-associated molecular patterns emission	Wang et al. (2018)
	Monocytes	Cyclic RGD-modified liposomes loaded with trefoil factor 3	Leukocytes for BBB crossing cRGD for brain targeting	Depression/olfactory bulbectomy rats	Excellent dual-brain-targeting and enhanced antidepressant effects of trefoil factor 3	Qin et al. (2015)
	Macrophages	Gold-silica nanoshells	Macrophage for BBB crossing	Brain metastases of breast cancer	Active targeting to brain metastases	Choi et al. (2012)
	Macrophages	DOX-loaded PLGA nanoparticles	Macrophage for BBB crossing DOX-loaded nanoparticles for tumor therapy	Glioma	Higher brain tumor distribution of nanoparticles and enhanced anti-glioma effect with prolonged survival	Pang et al. (2018)
	Macrophages	Nanocapsule consists of a Dox–silica complex	Macrophage for chemotactic migration Nanocapsule for controlled Dox release and cancer cell killing	Glioma	Efficient tumor growth suppression and minimal systematic toxicity	Zhang et al. (2018)
	Macrophage-derived exosomes	SPIONs and curcumin	Exosomes for BBB crossing Neuropilin-1-targeted peptide for glioma targeting SPION-mediated magnetic flow hyperthermia Curcumin for potent synergistic antitumor therapy	Glioma	Remarkable synergistic anti-glioma effect between MFH and Cur and extended survival of tumor-bearing mice	Jia et al. (2018)
	Macrophage-derived exosomes	Edaravone	Exosomes for brain targeting and improving bioavailability of drug Edaravone for neuroprotection	Ischemic stroke/permanent MCAO rats	Improved pharmacokinetic parameters and enhanced the bioavailability of edaravone Efficient delivery into the ischemic brain and enhanced neuroprotective effect	Li et al. (2020a)
	Macrophage	Polymer backpacks of catalase	Macrophage for BBB crossing Catalase for attenuating free radicals production	LPS-induced encephalitis mice model	Recruitment of macrophages with the attached backpacks to the inflamed brain Attenuated oxidative stress from activated microglia	Klyachko et al. (2017)
	Macrophage-derived exosomes	BDNF	Exosomes for BBB crossing BDNF for depression treatment	LPS-induced encephalitis mice model	Increased concentration of BDNF in inflamed brain	Yuan et al. (2017)
	Macrophage-derived exosomes	Catalase	Exosomes for BBB crossing and preserving drug during circulation Catalase for neuroprotection	6-OHDA mice PD model	Enhanced neuroprotective effects against neurodegeneration	Haney et al. (2015)
	Macrophage-derived exosomes	Curcumin	Exosomes for BBB crossing Curcumin to prevent neuronal death	OA-induced AD murine models	Inhibited Tau phosphorylation, enhanced neuroprotection in AD mice and reversed cognitive dysfunction	Wang et al. (2019)
	Neutrophil	PTX-loaded liposomes	Neutrophil for BBB penetration PTX-loaded liposomes for killing residual tumor cells and inhibiting tumor recurrence	Postoperative glioblastoma by open-window technique	Enhanced BBB penetration, slowed recurrent growth of tumor and improved survival rates	Xue et al. (2017)
	Neutrophil-derived exosomes	DOX	Neutrophils for inflammatory chemotaxis and BBB-crossing	Glioma	Efficient suppress of tumor growth and prolong survival	Wang et al. (2021)
	Neutrophils	Cross-linked DGL nanoparticles containing cis-aconitic anhydride-modified catalase	Neutrophil-mediated inflammatory migration for BBB penetration and ischemic brain targeting Catalase for ischemic stroke treatment	Ischemic stroke/MCAO mice	Reduced the infarct volume and inhibited of ROS-mediated apoptosis	Zhang et al. (2017)
	Neutrophils and monocytes	cRGD liposomes loaded with edaravone	Neutrophils and monocytes for brain targeting Edaravone for ischemia treatment	Ischemic stroke/MCAO mice	Enhanced protection against ischemic injury	Hou et al. (2019)
	Neutrophil membrane-derived nanovesicles	Resolvin D2	Neutrophil membrane-derived nanovesicles for inflamed brain targeting Resolvin D2 mitigate neuroinflammation	Ischemic stroke/ischemia and reperfusion mice	Prevent neurological damage during reperfusion for ischemic stroke therapy	Dong et al. (2019)
	Neutrophil membrane	Mesoporous Prussian blue nanozyme	Neutrophil membrane camouflage for BBB penetration and inflamed brain targeting Nanozyme induce microglia M2 polarization and neuron protection	Ischemic stroke/transient MCAO mice	Relieved ischemic damage and improved neurological function	Feng et al. (2021)

6-OHDA: 6-hydroxydopamine; AD: Alzheimer’s disease; BBB: blood–brain barrier; BDNF: brain-derived neurotrophic factor; DGL: dendrigraft poly-l-lysine; DOX: doxorubicin; LPS: lipopolysaccharide; MCAO: middle cerebral artery occlusion; OA: okadaic acid; PD: Parkinson’s disease; PLGA: poly(lactic-co-glycolic acid); PTX: paclitaxel; RGD: Arg-Gly-Asp-d-Tyr-Lys; SPIONs: superparamagnetic iron oxide nanoparticles.

## 2. Challenges of brain drug delivery

### 2.1. Stability during circulation

For blood-to-brain drug delivery, unexpected alteration of the drug or nanoparticles may occur during circulation due to complex biological components in blood flow. Drugs are usually cleared quickly from circulation. The fate of drugs or nanoparticles is hard to predict after phagocytosis, clearance, degeneration, or protein corona formation (Barbero et al., 2017; Chinen et al., 2017; Fromen et al., 2017). Also, degradation products may cause unwanted toxicity, thus limiting potential clinical applications. Thus, a protective carrier for drugs or nanoparticles for improving stability and facilitating the traveling from blood to brain is of importance for brain drug delivery. For nano-drug delivery systems, it should be noted that the formation of protein corona would make foreign objects, especially synthetic materials, more recognizable by the mononuclear phagocyte system (Bertrand et al., 2017). Improving the stability of drug by delivery systems during circulation is the first step for brain drug delivery.

### 2.2. BBB

The BBB is the primary challenge for brain drug delivery. BBB strictly limits the transcellular movement of drugs and nanoparticles from blood to brain (Gao, 2016). Macromolecules, small-molecules, proteins, antibodies, and nucleic acids are restricted by the BBB, which is a physical vascular barrier composed of brain endothelial cells, pericytes, and astrocytes, connected through tight junctions (Arvanitis et al., 2020; D'Souza et al., 2021). Changing characteristics or using delivery vehicles to breach the BBB has been considered more feasible than changing the permeability of BBB (Haumann et al., 2020). Penetration of BBB includes two process: recognition of receptors and intracellular transport (Ruan et al., 2021). Biomimetic drug delivery systems, such as cell or cell membrane coated nanotechnology can achieve BBB penetration by taking advantage of the natural delivery process such as energy/nutrition supply, chemotactic, or recruiting effects.

### 2.3. Targeting lesion spots

After entering the brain, targeting the specific site is also difficult (Gao, 2017). For brain diseases therapy, a minimal effective concentration of drug at the lesion spot is required for exerting desirable therapeutic effect. Drugs usually lack targeting effect, but drug delivery systems, especially biomimetic vehicles can alter the drug biodistribution and target the lesion spot by the endogenous driving force or intrinsic binding properties, such as cell-based vehicles or peptide ligands modified vehicles (Chen et al., 2020). Targets of diseased cells may be different, the discrimination ability of delivery systems to normal cells and diseased cells requires the understanding of the pathophysiology of the brain disease.

### 2.4. Cell internalization and drug release

To achieve therapeutic effects, for many drugs, they need to enter cells and reach subcellular targets. Cellular internalization of nanoparticles or drugs may involve both active transport and passive transport (Vacha et al., 2011; Russell et al., 2018; Anoop et al., 2020). After internalization, drug release is highly required to exerting therapeutic effect. Facilitating cellular uptake and achieving rapid and sufficient drug release are therefore important for the treatment of brain diseases.

## 3. Natural vehicles in blood

### 3.1. Erythrocytes

Erythrocytes, or red blood cells (RBCs), are biconcave-shaped blood cells without nucleus and organelles. Erythrocytes are the major components of blood cells, with a long life span. Erythrocytes have a high surface/volume ratio for efficient supply transport (Hamidi & Tajerzadeh, 2003). The cargo delivery ability of erythrocytes has been extensively explored (Bhateria et al., 2014). Until recent years, erythrocytes have been used for brain drug delivery (Xia et al., 2019). Several properties of erythrocytes enabled erythrocytes-based biomimetic vehicles as suitable brain delivery systems: (1) easy to obtain; (2) long circulation and life span; (3) uniformed size and shape size. Erythrocyte-based vehicles are versatile carriers with high engineering potentials and can be used for the delivery of various agents (Yoo et al., 2011).

### 3.2. Platelets

Platelets are also anucleate, discoid-shaped cells. Compared to erythrocytes, platelets are less in population and are small in size with a shorter life span (Koupenova et al., 2017). Blood platelets engaged actively in immunity, inflammation, and thrombosis (Koupenova et al., 2018). Platelet aggregation is of importance for hemostasis. In recent years, platelets have gained the great interest for drug delivery due to remarkable vehicle properties: (1) high storage and trafficking capacities (Banerjee & Whiteheart, 2017); (2) natural targeting and adhesion capacities (Induruwa et al., 2018; Wei et al., 2018). For brain drug delivery, platelets-based vehicles hold great potentials for brain ischemic stroke therapy for its natural targeting ability to the damaged blood vessel (Shi & Montgomery, 2010; Lu et al., 2019).

### 3.3. Leukocytes

Leukocytes are a group of immune cell subpopulations, including neutrophils, eosinophils, basophils, monocytes, and lymphocytes. Leukocytes are guardians of the body and have distinct physicochemical and biological characteristics. Leukocytes are the most widely explored drug delivery systems for their (1) adhesive ability (Liu et al., 2021), (2) migration and chemotaxis ability under diseased status (Kameritsch & Renkawitz, 2020), and (3) high loading capacity and efficient cargo convey (Huang et al., 2018). Leukocytes-based vehicles, for their inherent recruitment property, are especially suitable for drug delivery to brain diseases with inflammation.

## 4. Application of blood cell-based biomimetic delivery systems

### 4.1. Brain tumors

Brain tumors are aggressive in the central nervous system with high mortality (Barnholtz-Sloan et al., 2018). Currently, there is no curative treatment option for brain tumors and the five-year survival rate of patients with glioblastoma multiforme (GBM), a malignant subtype of brain tumor, is less than 6% (Ostrom et al., 2016). The major difficulty for brain tumor drug treatment is that the BBB hindered effective drug delivery to the tumor site (D'Amico et al., 2021). Also, the brain tumor shows a highly immunosuppressive microenvironment, which attenuates the immune responses to tumor cells, making their condition even worse (Gangoso et al., 2021). In addition, because of its high intratumoral heterogeneity, the brain tumor has a therapeutic resistance to many drugs and a high recurrence rate (Piper et al., 2021). Hence, the development of vehicles for crossing BBB and tumor targeting is of importance for brain tumor therapy.

#### 4.1.1. Leukocytes-based vehicles for brain tumor drug delivery

For the BBB penetration and tumor-infiltrating ability, leukocytes have first been explored for brain tumor drug delivery. Monocytes can also be used for the delivery of drug-loaded nanoparticles. Wang et al. developed Nano-DOX, composed of DOX attached to nanodiamonds (4–5 nm) with polyglycerol surface-coating, which improved the water-solubility of the drug (Zhao et al., 2014). Further, they connected cyclic tripeptide (l-arginine, glycine, and l-aspartic acid, RGD), which binds specifically to the integrin receptor avβ3 overexpressed on glioblastoma cells, to the nanodiamond to form the nanosystem. Monocyte-mediated delivery of the nanosystem successfully crossed the endothelial barrier and infiltrated the GBM spheroids *in vitro* (Wang et al., 2018).

Macrophages, originated from monocytes, have also been used for brain tumor delivery. Macrophages are major components of cell mass in solid tumors (Yang et al., 2020). Macrophages can be recruited into the tumor site by chemotaxis such as CCL2 (chemokine ligand 2) and CXCR4 (chemokine receptor 4). Macrophages, like monocytes, can cross the BBB and may be able to infiltrate the tumor. Brain metastasis is prevalent in subtypes (triple-negative and HER2^+^) of breast cancer (Zimmer et al., 2020; Lv et al., 2021). Choi et al. first reported that macrophages-mediated delivery of nanoparticles for brain delivery. They fabricated gold-silica nanoshells (66 ± 2 nm) and loaded them into macrophages. They found that macrophages could be used for effective brain delivery of nanoparticles while the BBB is intact (Choi et al., 2012). Macrophages can also be used for delivery of other types of nanoparticles for glioma therapy. Pang et al. incorporated DOX-loaded PLGA nanoparticles (156.9 ± 7.1 nm) into M1 macrophages. They found that the incorporation of nanoparticles did not impact the tumor-homing property of macrophages. Drug-loaded nanoparticles remained stable during systemic circulation via macrophages-mediated delivery with increased accumulation at the glioma site. Cellular interaction between macrophages and tumor cells facilitated the deep penetration of drug-loaded nanoparticles into tumors. DOX and M1 macrophages induced synergic apoptosis effects for glioma growth inhibition (Pang et al., 2018). To reduce drug release during cell-mediated delivery, Zhang et al. fabricated Dox–silica nanocomplexes (28.4 ± 3.4 nm) as nanocapsules by filling DOX and tetraethyl orthosilicate. Nanocapsules could be uptake effectively by macrophages, but minimally affect cell migration at early hours (6–12 h). This strategy increased the delivery efficiency and provided extra time for macrophages to arrive at the tumor site. Also, reduced drug release caused little systematic toxicity after intravenous administration (Zhang et al., 2018). Neutrophils also have intrinsic ability to penetrate BBB and could be used for brain tumor targeting. Xue et al. developed paclitaxel (PTX)-loaded liposomes and used neutrophils for targeting residual tumor cells after GBM surgery (Xue et al., 2017). Neutrophil carrying liposomal PTX realized triggered release to the remaining tumor cells and suppressed the recurrence of tumor growth.

In addition to living cells, cell-derived nanovesicles such as exosomes are also promising and maybe next-generation vehicles for advanced brain drug delivery. Inspired by the intrinsic inflammatory chemotaxis and excellent BBB-crossing capability of neutrophils, Wang et al. loaded DOX into neutrophil derived-exosomes (NEs-Exos) for glioma treatment. They reported that NEs-Exos penetrate the BBB rapidly and target the tumor site after intravenous injection. NEs-Exos loaded with DOX exhibited significant antitumor efficacy and prolonged the survival of glioma-bearing mice (Wang et al., 2021). Exosomes have a strong cargo-loading capacity, and the delivery potentials of exosomes can be enhanced by loading nanoparticles. Jia et al. developed superparamagnetic iron oxide nanoparticles (SPIONs) and curcumin dual-loaded macrophage-derived exosomes and conjugated the exosome membrane with neuropilin-1-targeted peptide (RGERPPR, RGE) to further improve the glioma-targeting ability. After administrated to orthotopic glioma-bearing mice, they found that the engineered exosomes successfully crossed the BBB and showed dual functions of tumor imaging and synergistic tumor therapy by SPION-mediated magnetic flow hyperthermia and curcumin-mediated therapy (Jia et al., 2018).

#### 4.1.2. Erythrocyte-based vehicles for brain tumor drug delivery

Long life span and stable circulation ability of erythrocytes can be used to improve the blood circulation of drugs and nanoparticles with low immunogenicity. As erythrocytes are anuclear, cell membranes are often extracted to coat nanoparticles for effective biomimetic drug delivery. But for brain targeting, engineering modification of the membrane is generally required for brain tumor therapy.

^D^CDX peptide, derived from candoxin, has shown BBB crossing ability for high binding affinity with nicotinic acetylcholine receptors (nAChRs) expressed on the surface of brain endothelial cells (Wei et al., 2015). Chai et al. developed DOX-loaded PLGA nanoparticles (average size around 95 nm) coated with erythrocyte membranes surface-modified with CDX peptides for brain targeting. The complex biological functions of erythrocytes were retained in membranes (Chai et al., 2017). This biomimetic delivery system successfully crossed the BBB exhibited improved therapeutic efficacy in glioma treatment and significantly reduced systemic toxicity. Further, based on the advantages of erythrocyte-membrane coating of nanoparticles, this research group developed docetaxel nanocrystals (average size around 70 nm) coated with erythrocyte-membrane surface-modified with c(RGDyK) tumor-targeting ligand. The erythrocyte-membrane coating improved the stability of drug nanocrystal particles (Chai et al., 2019). This delivery strategy is highly biocompatible with decreased side effects. Increased drug accumulation at the glioma site and enhanced treatment efficacy was observed after intravenous administration of the engineered cell membrane coated drug nanocrystal system.

Erythrocyte membrane coating can also improve the stability of the nucleic acid drug. In a recent study, Gao et al. designed a miR155-bearing nanogel (average size around 109 nm) with erythrocyte membrane-coating and functional M2pep peptides and HA2 peptides modification to mimic a virus structure. The membrane-coated nanoparticle was stable with prolonged circulation time. M2pep peptide modification endows the M2-microglia and macrophage targeting ability and HA2 peptide promotes fusion of membranes of erythrocyte and endosome. miR155 successfully delivered to the tumor site and entered the cytoplasm of macrophages and microglia and shifted their pro-invasive M2 phenotype to anti-tumor M1 phenotype for GBM immunotherapy (Gao et al., 2021).

### 4.2. Ischemic stroke

Stroke is a lethal disease and a leading cause of human death with high prevalence and disability (Hurford et al., 2020). Ischemic stroke is a primary type of stroke. Ischemic stroke is often caused by thrombosis or embolism which results in a severe reduction of blood flow and deficit of oxygen. During an ischemic stroke cascade, insufficient endogenous antioxidants fail to detoxify excessive reactive oxygen species (ROS) entities, which would react with complex materials, including lipids, proteins, and nucleic acids, in blood vessels of the brain (Chouchani et al., 2014). This process subsequently recruit abnormal inflammatory cells and then lead to cell death and consequently brain injury and infarction (Song et al., 2021).

Theoretically, thrombolytic drugs are ideal for treating cerebral ischemic diseases. However, the treatment time window is too narrow (normally <4.5 h) so that most patients are not able to receive thrombolysis and may suffer a brain injury (Khandelwal et al., 2016). After the time window of thrombolysis, neuroprotective agents such as antioxidants can be used to reduce the disease progression (Patel & McMullen, 2017). Antioxidants could be beneficial for ischemic stroke therapy by clearing clots and ROS, but little advancement was observed in trials. Reasons for the failure of antioxidants for ischemic stroke therapy may attribute to different pharmacokinetic profiles or BBB, which is challenging for clinical translation of antioxidant therapy in stroke. Hence, the development of drug delivery systems that can target lesion spots of ischemia in the brain is of importance for reversing the cascade and preventing stroke.

#### 4.2.1. Leukocytes-based vehicles for ischemic stroke drug delivery

In response to the neuroinflammation during ischemic stroke in the brain, leukocytes are recruited to the lesions spots. Utilizing this pathophysiological feature by engineering leukocytes for targeted drug delivery offered a unique opportunity for ischemic stroke therapy.

Catalase is a promising neuroprotectant that can convert hydrogen peroxide, a typical ROS in brain ischemia, to water and molecular oxygen with high efficiency. Under hypoxic conditions, catalase can exert a neuroprotection effect by serving as an alternate source of oxygen. Zhang et al. developed a nanosystem called *cl* PGP-PEG-DGL/CAT-Aco consisted of cross-linked dendrigraft poly-l-lysine containing cis-aconitic anhydride-modified catalase and modified with PGP tripeptide with high affinity to neutrophils. During circulation, the nanosystem would be selectively phagocytosed by neutrophils, which also protect the nanosystem during traveling, and then reach the ischemic spots via inflammatory tropism of neutrophils (Zhang et al., 2017). High targeting efficiency to the diseased sites and therapeutic efficacy for triggered release of the protein-based drug delivery system was observed. The therapeutic outcome of cerebral ischemia was greatly improved in MCAO mice. Similarly, Hou et al. exploited monocytes and neutrophils for delivery of edaravone, another neuroprotective agent that can scavenge free radicals to protect neuronal cells in neuroinflammation (Watanabe et al., 2008; Lapchak, 2010), to ischemic subregions and target cells to alleviate ischemic injuries. Liposomes loaded with edaravone were developed and engineered with cRGD (cyclo (Arg-Gly-Asp-d-Tyr-Lys) peptide for selective binding to integrin a_v_b_1_ highly expressed on monocytes and neutrophils surface to trigger internalization. Edaravone-loaded liposomes modified with cRGD were taken up to the lesion spots in the brain and protect against ischemic stroke (Hou et al., 2019).

Leukocytes-derived nanovesicles can also be used for drug delivery while retaining inflammation recruitment and BBB-crossing properties. Membrane coating and nanovesicles both possess highly complex biological functions similar to their cell of origin. The interaction between neutrophils and endothelial cells in brain blood vessels mediated cellular responses such as overproduction of radical oxygen species plays important role in ischemia/reperfusion damage (Becher et al., 2017). Resolvin D2 (RvD2), derived from docosahexaenoic acid, can induce the generation of nitric oxide to decrease leukocyte–endothelial cell interactions and reduce cytokine production (Spite et al., 2009). Dong et al. developed neutrophil membrane-derived nanovesicles, by disrupting cells and ultracentrifugation, for targeting ischemic sites at the brain to deliver RvD2 to endothelium to inhibit endothelial activation, prevent cytokine release and further neutrophil recruitment (Dong et al., 2019). This delivery strategy successfully prevented brain injury in the ischemia/reperfusion mice model. Focusing on ROS in ischemic stroke, Feng et al. utilized the inflammation-recruiting effects of neutrophil and fabricated neutrophil membrane-coated mesoporous Prussian blue nanozyme for targeted long-term therapy of ischemic brain damage by scavenging ROS to reduce neutrophil recruitment, shift microglia polarization, and decrease the apoptosis of neuronal cells (Feng et al., 2021).

Exosomes secreted by macrophages can breach the BBB and release the cargo at the inflamed site in the brain. Li et al. loaded edaravone into macrophage-derived exosomes for protecting neuronal cells and promoting the polarization of microglia from M1 to M2. The bioavailability of edaravone was improved greatly with a prolonged half-life in exosomes. Intravenous injection of edaravone-loaded exosomes targeted the ischemic brain and exhibited significantly free radical scavenging effects via exosomes-mediated delivery (Li et al., 2020a).

#### 4.2.2. Erythrocyte/platelet-based vehicles for ischemic stroke drug delivery

Dissolving thrombus clog is beneficial for the treatment of ischemic stroke. Recombinant tissue plasminogen activator (rtPA) has been approved for the clinical thrombolytic treatment of ischemic stroke, but the poor affinity of the drug to the clog in the brain limited its efficacy (Rochette et al., 2003). Besides, dissolving thrombus clog needs to be cautious as ischemia/reperfusion injury may occur. To solve the dilemma, Xu et al. developed a thrombin-responsive platelet biomimetic nanoplatform for dual-drug delivery for ischemic stroke treatment. ZL006e (5-(3,5-dichloro-2-hydroxybenzylamino)-2-hydroxybenzoic acid) is a neuroprotective agent, which can selectively block the ischemia-induced PSD-95/nNOS coupling (Zhou et al., 2010). ZL006e-loaded acetal-modified dextran (m-dextran) polymer nanoparticles were coated with platelet membranes and rtPA was decorated on the platelet membranes. Further, to achieve the thrombin-triggered release of rtPA and enhance BBB penetration of the system, a thrombin-cleavable peptide and a Tat cell-penetrating peptide were introduced. This nanoplatelet system, with prolonged circulation time and BBB penetration ability, exhibited significant efficacy of ischemic stroke by recovering the cerebral blood supply and protecting neurons of ischemic penumbra simultaneously (Xu et al., 2019).

Inspired by the inherent properties of platelets in targeting adhesion to injured vasculature, Li et al. first fabricated nanobubble (131.43 ± 19.84 nm) by repeated freeze-thawing and sonication of membrane vesicles derived from platelets (Li et al., 2018). PNBs could naturally target brain occlusions from the onset of stroke due to the natural vessel adhesive components and glycoproteins. The nanobubble could target acute ischemic lesion spots in the brain and recanalize the microvasculature. Besides, accumulation and merging of nanobubbles at the lesion could provide real-time detectable ultrasound-enhanced signals for dynamic monitoring. Further, utilizing the targeting ability of platelets to damaged blood vessels, this research group fabricated a biomimetic drug delivery system (215.50 ± 8.05 nm) comprising l-arginine and γ-Fe_2_O_3_ magnetic nanoparticles in platelet membranes (Li et al., 2020b). This biomimetic nanocarrier can target thrombus actively and with the guidance of external magnetic, and then deliver l-arginine to ischemic lesion spots for producing nitric oxide to promote vasodilation and reduce aggregation of platelets and leukocytes to the blood vessel endothelial.

Erythrocytes have also been explored as brain drug delivery systems for salvaging ischemic stroke for its BBB crossing advantage and native stealth property. Oxygen supply and free radical scavenging are of significance for preventing brain injuries during ischemic stroke. Shi et al. engineered T7 peptide-modified erythrocyte membrane-coated manganous tetroxide nanoparticles as a versatile oxygen sponge for ischemic stroke treatment and preventing ischemia/reperfusion injury (Shi et al., 2020). With the help of T7 peptides, Mn_3_O_4_, an antioxidant mimic nanoenzyme, encapsulated in erythrocyte membranes, traversed the BBB, and efficiently enrich infarct areas. Before thrombolysis, this biomimetic nanoerythrocyte can protect neuronal cells by scavenging free radicals and generating oxygen; after thrombolysis, this biomimetic nanoerythrocyte can prevent ischemic/reperfusion injury by regulating oxygen influx for the storage property of Hb.

### 4.3. Other brain diseases

#### 4.3.1. Blood cell-based brain drug delivery vehicles for encephalitis therapy

Encephalitis is a brain inflammation disease often caused by infection (Mailles et al., 2017). The pathology of brain inflammation cascade is associated with increased migration of immune cells utilizing diapedesis and chemotaxis from blood to brain, crossing the BBB, which breaks down during brain inflammation. Klyachko et al. developed cellular backpacks (micron-scale in size and a few hundred nanometers in thickness) loaded with catalase and attached to the surface of macrophages without affecting the natural functions of the cell carrier (Klyachko et al., 2017). Backpacks loaded with catalase successfully crossed the BBB in lipopolysaccharide (LPS)-induced mouse model of encephalitis and attenuated oxidative stress of microglial cells in the brain. In another study, Yuan et al. utilized naïve macrophage-derived exosomes for brain delivery of brain-derived neurotrophic factor (BDNF) for treating encephalitis (Yuan et al., 2017). They observed that, in the presence of inflammation, the upregulation of intercellular adhesion molecule 1 (ICAM-1) on brain microvessel endothelial cells promoted uptake of macrophage-derived exosomes and facilitated their BBB crossing, and as a result, increased BDNF levels in inflamed brain.

#### 4.3.2. Blood cell-based brain drug delivery vehicles for depression therapy

Depression is a major public health burden (Chu et al., 2021). Qin et al. found that trefoil factor 3 (TFF3) has antidepressant effects in animal models, but the dose needs to be high for therapeutic application (Shi et al., 2012). To further increase the concentration of TFF3 in the brain, they developed cRGD-modified liposomes (cRGDL) with a high affinity to integrin receptors on monocytes (Qin et al., 2015). The monocyte-cRGDL (loaded with TFF3) complexes, with BBB-crossing and brain-targeting abilities, increased the brain distribution of TFF3 and exhibited enhanced antidepressant effects and behavioral response of TFF3 treatment.

#### 4.3.3. Blood cell-based brain drug delivery vehicles for Parkinson’s disease therapy

Parkinson's disease (PD) is an age-related and typical neurodegenerative disorder (Bloem et al., 2021). The prevalence of PD is growing fast (Broen et al., 2016). The pathology of PD is associated with inflammation, microglial activation, and overproduction of ROS in the brain (Kalia & Lang, 2015). Reduced levels of antioxidants such as redox enzymes, superoxide dismutase, and catalase have been reported in the brain of patients with PD (Abraham et al., 2005). This may cause oxidative stress and neurodegeneration in PD patients. In this regard, a successful brain delivery of catalase may be helpful for deactivating free radicals for PD therapy. Haney et al. encapsulated catalase into macrophage-derived exosomes to prolong blood circulation time and facilitate brain delivery, thereby improving anti-PD efficacy (Haney et al., 2015).

#### 4.3.4. Blood cell-based brain drug delivery vehicles for Alzheimer’s disease therapy

Alzheimer’s disease (AD) is a neurodegenerative disease with progressive memory loss, cognitive dysfunction, executive dysfunction, and behavioral changes (Scheltens et al., 2021). Mounting evidence demonstrated that AD is associated with extracellular inflammatory plaques formed by phosphorylated Tau protein (Canepa & Fossati, 2020). Inhibition of phosphorylation and aggregation of Tau is a potential therapeutic target for AD. The inhibition of GSK-3β-mediated phosphorylation pathway of Tau may alleviate AD progression (Lu et al., 2013). Curcumin, a diarylheptanoid, is able to regulate Tau phosphorylation and may be used for AD therapy (Okuda et al., 2016). But poor water solubility and low bioavailability limited the application of curcumin. To overcome the shortcomings of curcumin and explore its therapeutic effects on AD, Wang et al. treated macrophages with curcumin to fabricate curcumin-loaded exosomes with improved solubility, stability, and tissue bioavailability (Wang et al., 2019). More importantly, curcumin-loaded exosomes featured high BBB-crossing ability via receptor-mediated transcytosis. Curcumin-loaded exosomes successfully accessed brain tissues and inhibited Tau phosphorylation and recovered neuronal function in murine AD models.

## 5. Summary and perspective

Advancements in nanomedicine have shown significant advantages in both efficacy and safety and offered opportunities for targeted delivery of various therapeutic agents. However, brain drug delivery remains a major challenge as current nanomedicines have shown limited ability to cross the restrictive BBB (Ferraris et al., 2020). In recent years, bioinspired drug delivery systems are emerging for multifunction and excellent *in vivo* performance. For brain drug delivery, blood cell-based vehicles are attractive biomimetic candidates for their unique features such as high biocompatibility, long circulation, improved drug loading stability, BBB penetration, and potential targeting abilities. Blood cells can be used directly or for coating nanotechnology. Major components of blood cells, including erythrocytes, platelets, leukocytes, and their derivate vesicles have been used for drug delivery for various brain diseases.

Erythrocyte-based vehicles have probably the longest circulation ability and the best stability of drug loading and suitable for various drugs and nanoparticles. Erythrocyte membranes are outstanding carriers for improving pharmacokinetic profiles of drugs or nanoparticles. Erythrocyte membrane could be used to prevent macrophage phagocytosis and prolong blood circulation of nanoparticles and drugs (Gao et al., 2013). For brain tumor drug delivery, most studies used erythrocyte membranes for coating nanoparticles. However, erythrocyte-based vehicles may have less ability for BBB penetration and targeting. Peptides, such as RGD are often used to modify erythrocyte membranes to facilitate BBB penetration and improve their targeting ability. Besides, cell internalization of erythrocyte-based vehicles after reaching the targeting lesion may be another problem due to their intrinsic ‘self-recognition’ nature. A design for the membrane disruption should be considered. Besides, the high biocompatibility of erythrocytes may be affected after engineering and modifications.

Platelets are functional blood elements for hemostasis. Platelets are also stable during circulation and can target damaged blood vessels. These features of platelet also limited the therapeutic application. Abnormal activation of platelets may increase bleeding and thrombosis risks. Platelets are regulators of neuroinflammation and play important roles in the integrity of BBB (Brailoiu et al., 2018; Gao et al., 2020). Currently, for brain drug delivery, platelet-based vehicles are being used solely for ischemic stroke therapy as they may have limited BBB penetration ability under other brain diseased status. The use of platelet-based vesicles also has several limitations. Platelets have high reactivity and sensitivity. Activation of platelets may lead to undesired thrombosis or bleeding. Besides, once isolated *in vitro*, platelets often aggregate, therefore, their status and morphology after cargo loading should be checked before infusion.

Leukocytes, with subpopulations, are minor but versatile populations in blood. Leukocytes have intrinsic properties such as tumor targeting, inflammatory chemotaxis, and BBB penetration. Different subpopulations of leukocytes may be able to cross the BBB but have distinct functions, which affect their application of brain diseases. In response to the inflammation, neutrophils can traverse BBB with crosstalk to brain endothelial cells (Wu et al., 2018). For brain diseases associated with inflammation, neutrophils are often used for higher recruitment ability. However, for brain tumor drug delivery, most studies used macrophages or their derived vesicles for their tumor-targeting ability. Macrophage is a key immune cell type and could be recruited to inflamed brain in response to chemokines (Ye et al., 2018). For brain tumor therapy, chemotherapeutic agents were loaded into nanoparticles and then engineered with membrane coating or directly loaded into living cells. For cell-based drug delivery, there is an issue of drug leaking which would decrease the viability of cells then affect their delivery, despite there was report showing no release of encapsulated PTX from liposomes (Xue et al., 2017). In addition, although promising results of leukocytes and their derived vesicles as drug vehicles have been reported, there are risks of immune system overloading and aggravate-induced inflammation after additional import. Besides, quality control of leukocyte-based vehicles may be challenging for discrimination of subspecies. The leading of drugs in nanoparticles is another problem for cell-based drug delivery.

A growing number of studies have been using cell-derived nanovesicles for brain drug delivery. It has been reported that transcytosis may be the mechanism underlying the BBB-breaching process of exosomes (Morad et al., 2019). But there has been a doubt that whether exosomes could retain their naïve structure and cargo after BBB penetration. It is possible that exosomes are first uptake by brain endothelial cells and then be released, with their cargo, in brain. Further understanding of the interaction between nanovesicles and BBB will guide the development of brain drug delivery systems.

In summary, blood cell-based drug delivery systems hold great potentials for precision medicine of brain diseases in the future for their natural properties (Castro et al., 2021). To maximize therapeutic effects, suitable blood cell-based vehicles with appropriate engineering modification are encouraged according to the physiopathological process of the disease. A deep understanding of the biological properties of blood cell-based vehicles is of importance for precision and novel applications. For clinical translation of the blood cell-based drug delivery systems for brain drug delivery, cell source, large-scale production, standardized characterization, quality control, and comprehensive biological evaluations are major and urgent challenges that require further efforts of multi-field collaborations.
